# Platelet-Derived Growth Factor-Modulated Guided Tissue Regeneration with a Bioresorbable Membrane in Class III Furcation Defects: A Histometric Study in the Monkey

**DOI:** 10.3390/ma14092420

**Published:** 2021-05-06

**Authors:** Dietmar Weng, Lina Stapf, Matthias Kern, Ralf-Joachim Kohal

**Affiliations:** 1 Department of Prosthodontics, Propaedeutics and Dental Materials, School of Dentistry, Christian-Albrechts University, Arnold-Heller-Str. 16, 24105 Kiel, Germany; l.bruchmann@gmx.de (L.S.); mkern@proth.uni-kiel.de (M.K.); 2 Medical Center—University of Freiburg, Center for Dental Medicine, Department of Prosthetic Dentistry, Faculty of Medicine, University of Freiburg, Hugstetter Str. 55, 79106 Freiburg, Germany; ralf.kohal@uniklinik-freiburg.de

**Keywords:** class III furcation, guided tissue regeneration (GTR), platelet-derived growth factor (PDGF), animal study, histometric evaluation

## Abstract

It was the aim of this study to histometrically evaluate guided tissue regeneration (bioresorbable membrane plus bone mineral) (GTR) with or without platelet-derived growth factor (PDGF) in two different types of class III furcation defects (small keyhole defects and horizonal defects) in monkeys. In six cynomolgus monkeys, two types of class III furcation defects were created and allowed to chronify for 5 months in mandibular first and second molars. After a hygiene program the molars were assigned to GTR group (collagen membrane plus bovine bone mineral), PDGF group (collagen membrane plus bovine bone mineral plus PDGF), or negative control group (flap reposition only). Histologic sections were made after 7 months of healing and descriptive statistics were provided from the histometric parameters. Postoperative healing was uneventful despite marginal membrane exposures in the GTR and PDGF group. Bone regeneration of 23–35% of the original defect area was found in the two treatment groups. In none of the evaluated key parameters (formation of bone, root cementum, connective tissue, or epithelium) differences were detected between GTR and PDGF groups. However, the negative control teeth exhibited better bone regeneration than the treatment groups. The type of class III defect did not influence the regenerative outcome. Within the limits of this study PDGF was not able to enhance the histologic regeneration of class III furcation areas in monkeys compared to bone mineral enhanced GTR treatment regardless of the defect configuration. Membrane exposure during early healing might have influenced these outcomes.

## 1. Introduction

The treatment outcome of class III furcation defects has always been compromised due to its limited and unpredictable success. Whereas class I and II furcations can be handled reliably in most cases with a non-surgical [1] or surgical approach [2,3,4], the anatomy of the furcation area in general [5] and the architecture of a class III furcation defect involve unfavorable conditions for guided tissue regeneration (GTR) [6]. Especially the big distance between the areas to be regenerated and the remaining periodontal ligament cells as well as the associated exposure of the furcation entrance caused by soft tissue recession seem to be the main problems for successful GTR in class III furcations [7]. Various single and combined treatment modalities have been considered to regenerate class III furcations. Among them were expanded polytetrafluoroethylene (ePTFE) membranes [2,7,8,9,10,11,12,13,14], polylactide membranes [15,16], elastin-fibrin membranes [17], fibrin adhesive material [18], decalcified freeze-dried bone allograft [19,20,21], hydroxyapatite [22], and tricalcium phosphate [23]. None of them provided satisfying or predictable results for a closure of the class III furcation defects.

Additionally, growth factors have been introduced into the periodontal regenerative armamentarium [24]. One of them, platelet-derived growth factor (PDGF), showed significant enhancements in regeneration of periodontal defects [25,26,27,28,29]. Its strong chemotactic and mitogenic effect on periodontal ligament cells is considered the main reason for improved regenerative results [30]. PDGF is also released together with other cytokines following platelet activation. Therefore, it is one of the main growth factors underlying the effects of platelet concentrates. [31]. There is evidence that platelet concentrates are effective in enhancing periodontal wound healing [32]. Some research groups also recommended the combination of barrier membranes with PDGF so as to combine the effects of periodontal ligament fibroblast stimulation and prevention of epithelial cell ingrowth [29,30,33].

Therefore, the purpose of this study was to evaluate the effect of a bioresorbable barrier membrane in combination with a bone mineral graft with or without PDGF in class III furcation defects. Furthermore, the effect of the defect configuration on the regenerative results was tested.

## 2. Materials and Methods

In this study, six adult male cynomolgus monkeys (Macaca fascicularis) were used and treated according to the guidelines of the Animal Welfare Committee of the University of Texas at Houston, Health Science Center (Approval Number HSC-AW-98-073). General anesthesia was induced by ketamine i.m. (10–15 mg/kg) and maintained by gas intubation with 1.5–2 vol.% isoflurane. In addition, the anesthetic agent buprenorphine i.m. (0.01–0.02 mg/kg) was administered. Local injection of lidocaine 2% with epinephrine 1:50,000 into the surgical areas reduced hemorrhage.

**Figure 1:**
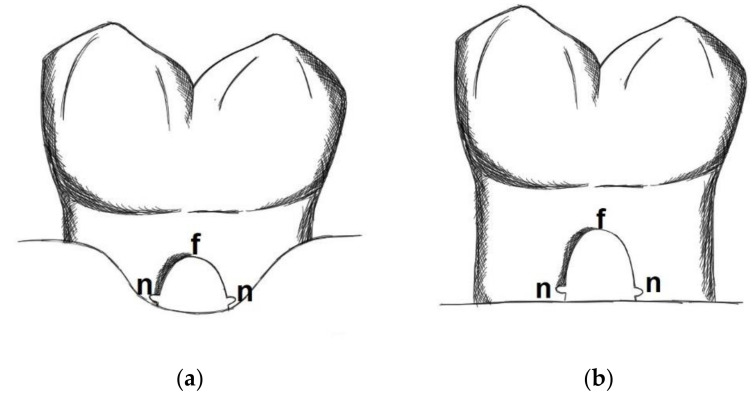
Small keyhole defect (**a**) and horizontal defect type (**b**). After chronification of the defects notches (n) were made with a round bur at the level of the bone crest at the apical end of the furcation (f).

After elevation of mucoperiosteal flaps on the buccal and lingual aspects, in both sides of the mandible class III furcation defects were created between the mesial and distal roots of the first and second molars. On one side of the mandible small keyhole defects of 2 mm × 2 mm were surgically created with round burs and chisels, whereas on the contralateral side the defects were not only slightly larger in size but also the bony attachment mesial of the mesial root and distal of the distal root was removed, thus producing kind of a circumferential horizontal bone loss. Similar kind of defects have been previously described by Pontoriero et al. [7] (Figure 1). The root surfaces were scaled and root planed meticulously to completely remove the periodontal ligament. To enhance plaque accumulation of the class III furcation defects and to prevent spontaneous wound healing during the following months, an impression material (Reprosil^®^, Dentsply Sirona, York, PA, USA) was brought into the furcation areas. The flaps were readapted and sutured back to their original position. For the following 5 months, no oral hygiene measures were taken to allow for heavy plaque formation and chronic inflammation of the sites. After the first 3 months of plaque formation, the impression material was removed from all mandibular molars. At month 5 the teeth were scaled supragingivally and an oral hygiene regimen was established three times per week (tooth brushing with pumice and chlorhexidine) for the following 4 weeks.

At month 6, buccal and lingual mucoperiosteal flaps were raised in the lower molars again. All defects were thoroughly debrided, scaled and root planed. In the furcation defects, root notches were made at the level of the bone crest inside the furcation area (Figure 1). The molars on each side were randomly assigned to either the GTR group (bioresorbable porcine collagen membrane Perio-Gide^®^ plus bovine bone grafting material Bio-Oss Collagen^®^, both from Geistlich Pharma AG, Wolhusen, Switzerland) or to the PDGF group (bioresorbable procine collagen membrane Perio-Gide^®^ plus bovine bone grafting material Bio-Oss Collagen^®^, both from Geistlich Pharma AG, Wolhusen, Switzerland, plus an experimental PDGF (rr-PDGF-BB, i.e., recombinant rat (*E. coli*—derived) PDGF BB)) or to the control group (no further treatment done). The GTR group, i.e., a combination of barrier membrane and bone graft, was considered the standard treatment (positive control) for periodontal defects, whereas the PDGF group represented the test group by adding the PDGF and using the bone graft as a carrier. Therefore, only a small number of teeth were added as negative controls. In both the small keyhole defects and the larger horizontal defects 5 teeth were assigned to the GTR group, 5 teeth to the PDGF group and 2 teeth to the control group. The bovine bone grafting material was mixed with saline 0.9% in the GTR group and additionally with the PDGF solution in the PDGF group, before it was brought into the furcation area. Membranes were adapted and sutured to the lingual and buccal aspects of the furcation entrance in the two test groups. The flaps were readapted without tension by releasing the periosteum and sutured over the defects.

The postoperative regimen consisted of anti-inflammatory and analgesic agents (buprenorphine and ibuprofen) to reduce postoperative swelling and pain, chlorhexidine rinses for the first 2 weeks after surgery (thereafter, tooth cleaning with chlorhexidine and pumice 3 times per week) and suture removal after 7–10 days.

The tissues and defects were allowed to heal for 7 months. At month 13 of the experiment the animals were anesthetized and sacrificed by exsanguination. The heads of the animals were fixed by vascular perfusion with 2% glutaraldehyde in 0.1 M sodium cacodylate buffer following a carotid artery cut-down procedure. Following this initial fixation, the mandibles were block-resected and immersed in half-strength Karnovsky’s fixative buffered to a pH of 7.4 with 0.02 M sodium cacodylate at 4 °C for 48 h. Subsequently, they were kept in 0.185 M sodium cacodylate buffer until processing for undecalcified ground sections according to the method of Donath & Breuner [34]. Thus, sections of approximately 30 μm in thickness were made from the molars and stained with toluidine blue solution. According to the embedding procedure and grinding angulation, for each tooth 1–4 sections were available for histometric analysis.

**Figure 2:**
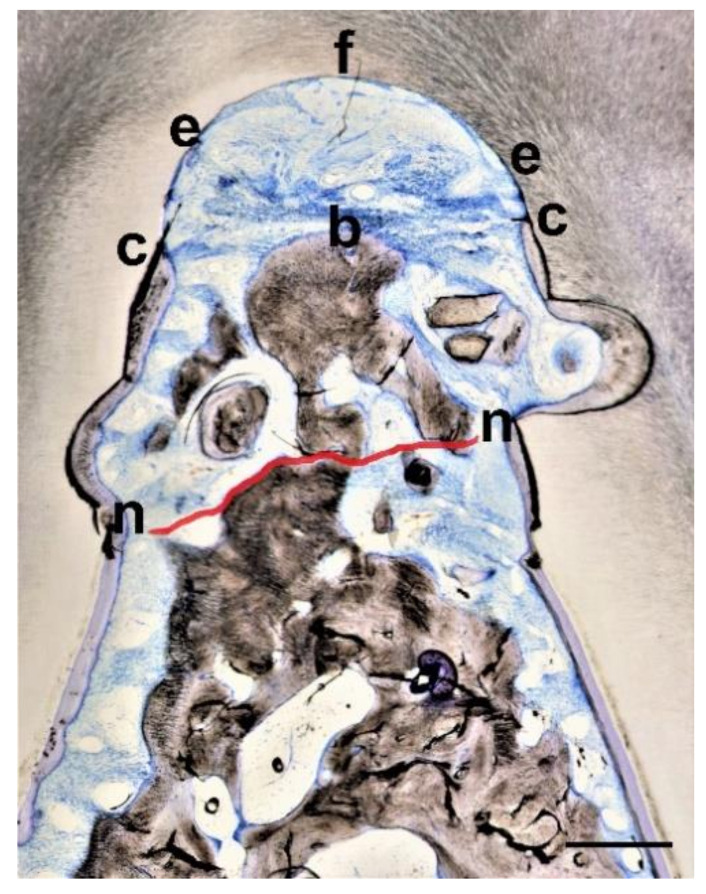
Sample histology with the histometric landmarks f (coronal end of furcation), n (apical end of notch), b (coronal end of newly formed bone), c (coronal end of newly formed cementum), and e (apical end of epithelium). Red line delineates old bone (below the red line) from newly formed bone (above the red line). Bar in lower right corner represents 0.5 mm.

Descriptive histology and histometric analyses were carried out by light microscopy evaluating the following parameters (Figure 2):

1. Defect height: Vertical distance between apical end of root notch (n) and coronal end of furcation (f)
2. New bone height: Vertical distance between apical end of root notch (n) and coronal end of newly formed bone (b)
3. New cementum height: Vertical distance between apical end of root notch (n) and coronal end of newly formed cementum (c)
4. New connective tissue height: Vertical distance between apical end of root notch (n) and apical end of epithelium (e)
5. Epithelial height: Vertical distance between apical end of epithelium (e) and coronal end of furcation (f)

Parameters 1–5 were measured separately along the distal and mesial root within the furcation and then a mean value between the two measurements was used as value for the respective section.

6. Defect area: Area outlined by a line between the apical ends of the notches and the root surfaces along the furcation.
7. New bone area: Area occupied by newly formed bone within the defect area
8. New cementum area: Area occupied by newly formed cementum within the defect area
9. New soft connective tissue area: Area occupied by newly formed soft connective tissue within the defect area
10. Epithelium area: Area occupied by epithelium within the defect area
11. Free bone graft area: Area occupied within the defect area by bone graft particles not embedded into new bone

Parameters 2–5 and 7–11 were expressed as percentages of parameter 1 and 6, respectively. From the available sections of a certain tooth a mean value per tooth was calculated. The statistical unit n for the average values and standard deviations was the tooth, i.e., total n was 24. The histologic and histometric assessment was carried out blind with regard to the treatment which has been done. Descriptive statistics, i.e., means and standard deviations (Microsoft Excel), a Kruskal–Wallis test to compare between the 3 treatment groups, and a Mann–Whitney test to compare within the treatments groups between the 2 different defect sizes (www.statskingdom.com (accessed on 24 April 2021)) were used to present and analyze the data (significance level set at 0.05).

## 3. Results

### 3.1. Clinical Findings

Healing was uneventful in general. Marginal exposures of the collagen membranes of 1 to 1.5 mm on the buccal and lingual sides were observed during the first 3 postoperative weeks in all test defects (PDGF and GTR groups). Due to the resorption of the collagen the exposed membranes dissolved after approximately 4 weeks. Slight gingival recession was noted thereafter in those areas.

### 3.2. Histological Findings

**Figure 3:**
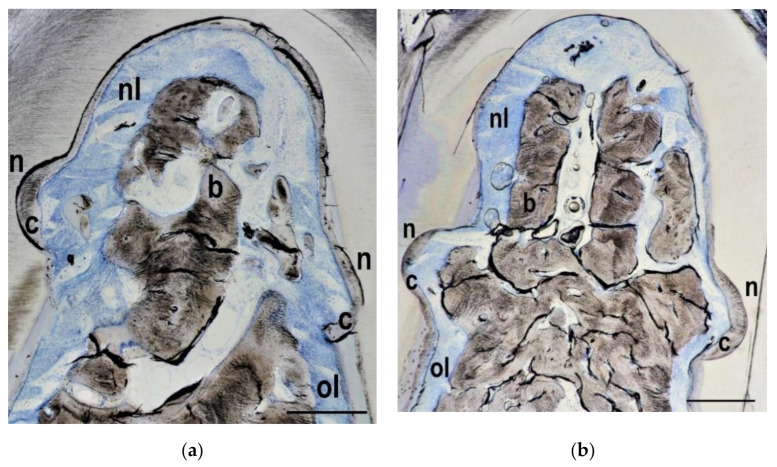
Control group histology from the keyhole (**a**) and the horizontal (**b**) defect types. The notches (n) on both sides as well as the whole defect area have been covered with a layer of new cementum (c) onto the formerly contaminated and debrided root surface. New bone (**b**) has grown into the furcation area and occupied approximately two thirds of the defect height. New periodontal ligament (nl) has formed coronally to the original periodontal ligament (ol). Bar in lower right corner represents 0.5 mm.

**Figure 4:**
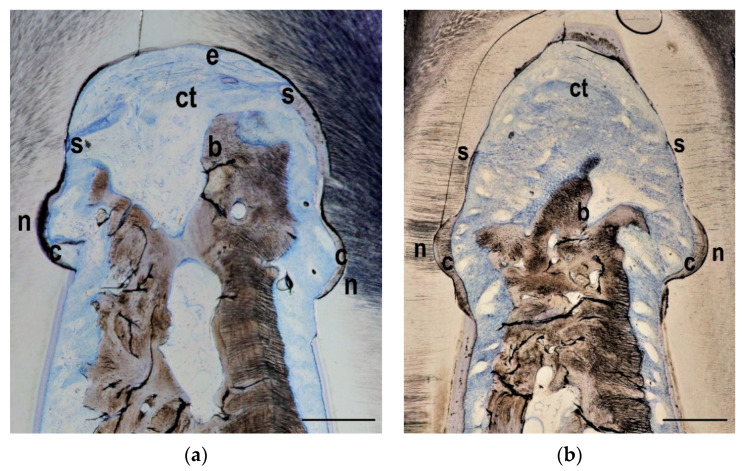
GTR group histology from the keyhole (**a**) and the horizontal (**b**) defect types. Whereas the notches (n) on both sides of the defect have still been covered with a layer of new cementum (c), the cement formation has stopped (s) coronal to the notches and has not covered the whole furcation roof. Some new bone (**b**) has grown into the furcation area and occupied approximately half of the defect height. Connective tissue (ct) and epithelium (e) have formed in the coronal part of the furcation. Bar in lower right corner represents 0.5 mm.

**Figure 5:**
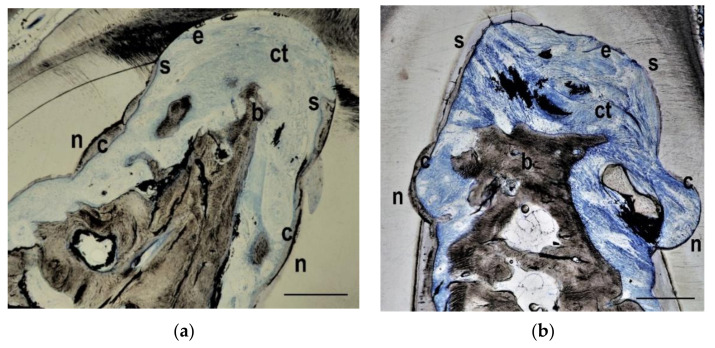
PDGF group histology from the keyhole (**a**) and the horizontal (**b**) defect types. Similar to the histologies of the GTR group, the notches (n) on both sides of the defect have been covered with a layer of new cementum (c), but the cement formation has stopped (s) coronal to the notches and has not covered the whole furcation roof. Again, some new bone (**b**) has grown into the furcation area and occupied approximately half of the defect height. Connective tissue (ct) and epithelium (e) have formed in the coronal part of the furcation. Bar in lower right corner represents 0.5 mm.

In 23 out of 24 teeth new bone formation was visible within the furcation areas. The overall average bone height in the two treatment groups and in the control group was approximately 60% of the defect height, with markedly better regeneration in the control teeth. Similar observations were made when the bone area was examined: Overall 35% of the original defect area was filled with bone, but again the control teeth showed more pronounced regeneration than the teeth from the test groups. New cementum formation was visible in all teeth. Sometimes the new cementum formation was not continuous but interrupted along the formerly contaminated root surface. New cementum was different from the original acellular extrinsic fibres cementum and consisted of cellular intrinsic fibres cementum. Soft connective tissue and/or epithelium were present in all specimens. Ankylosis has not been detected in any of the specimens. Exemplary histologies for each group are presented in Figure 3, Figure 4 and Figure 5.

### 3.3. Histometrical Measurements

Altogether 65 histological sections were suitable for histometrical evaluation, the number of sections for an individual tooth ranging from 1 to 4. In Table 1 (small keyhole defects) and Table 2 (horizontal defects) the individual results can be seen. Although the total amount of regenerated bone and cementum height and area was encouraging, there was a clear tendency regarding the regeneration with new bone and new cementum that both GTR and PDGF groups were not different from each other and worse than the untreated controls (*p* > 0.05 between the 3 treatment groups in all measured parameters). Furthermore, no influence of the defect configuration was detectable (*p* > 0.05 between defect configurations in all measured parameters within the same treatment group).

## 4. Discussion

Periodontal regeneration in furcation class I and II defects has been documented clinically and histologically well in the literature and seems to be quite predictable. This is not the case in class III furcation defects [35]. The number of studies examining animal models for histologically regenerating this kind of defect is limited, especially in higher animals. Pontoriero et al. [7] have shown that with ePTFE membranes complete regeneration of chronic class III furcations in dogs is possible. However, the defect should not be bigger than 2 × 2 mm (small keyhole defect) and membrane exposure should not occur. If the defects were bigger (3 × 3 mm, i.e., large keyhole defects, or horizontal defects), regeneration with new bone and cementum was not achieved because membrane exposure occurred and flap recession, exposing the through-and-through area, was the consequence. In our monkey model the furcation class III defects were resembling closer to furcations in humans than in a dog model. The small keyhole defects measured 2 × 2 mm in size, i.e., identical to Pontoriero et al. [7] despite the smaller molar sizes of the monkey, and the larger horizonal defects simulated a more progressed horizonal bone loss exposing the furcation entrances.

Park et al. [29] showed in a beagle dog study without a bone graft underneath the membranes that PDGF in addition to GTR had a positive influence on regeneration of class III furcation areas compared to GTR alone. They examined 6 animals after 5, 8, and 11 weeks (2 animals at each time point, each with 4 treated teeth; 2 teeth had GTR plus liquid vehicle only, 2 teeth had GTR plus PDGF), and reported that there were no differences after 5 weeks. However, after 8 weeks the PDGF group had more bone fill and new periodontal ligament in the furcation areas than the GTR group. At 11 weeks the GTR group had almost the same bone fill as the PDGF group. Therefore, the authors concluded that the initial healing process progressed faster in the PDGF group. However, untreated controls were not available.

The results of this monkey study were not able to demonstrate a positive effect of the added growth factor PDGF, but might stress instead the importance of the direct postoperative healing phase [36,37], especially as far as barrier membrane exposure is concerned. Membrane exposure has been shown to worsen the results after periodontal regenerative surgery [38,39]. Colonization with microorganisms and faster membrane degradation are factors interfering with undisturbed proliferation of new ligament cells. The limited number of negative controls in our study showed better regeneration in the furcation areas than the two treatment groups in which barrier membranes were applied according to GTR principles. Furthermore, the addition of PDGF to the bone graft mineral was not able to enhance regeneration in the PDGF group, irrespective of the type of class III furcation. Even limited exposure of resorbable barrier membranes can negatively influence the regenerative results, as a recent retrospective study in humans has demonstrated [40]. The unfavorable defect morphology of a class III furcation defect predisposes to bacterial contamination and, possibly, local infection during the early healing phase. One might even speculate, whether a barrier membrane (although it is fundamental part of any classical GTR procedure) is counterproductive in challenging defects such as class III furcation areas, because any exposure might accelerate the access of bacteria to the regenerative space and thus compete with the added benefit of a growth factor. Not even resorbable membranes (compared to non-resorbable membranes) and their faster degradation properties after exposure might compensate for such factors negatively influencing the regenerative result. In addition, differences can be expected whether the exposure of a resorbable barrier membrane leads to defect exposure or not [41]. Keeping the flap above the barrier membrane has always been a challenge in regenerative surgery of class III furcation defects. Nevertheless, it is probable that barrier membrane exposure rates are reduced in humans, since they usually follow postoperative instructions of wound care compared to animals.

Prolonged contamination time of 3 months with the impression material in the furcation area could be another reason for the reduced regenerative results in the test groups of our monkey model. Pontoriero et al. [7] used in their dog model a shorter contamination time of 3 weeks with impression material. The longer contamination time was chosen to adapt the chronification period after surgical defect creation to a more clinically realistic scenario.

Furthermore, one might speculate (upon comparing the two test groups) that a beneficial effect of PDGF might have worn off after 7 months and would have possibly been detectable after a shorter observation time. On the other side, clinical (and radiographical) re-assessments after regenerative periodontal surgery are usually performed 6 months after surgery in order not to disturb wound healing in a clinical setting with patients. Therapeutic approaches not lasting longer than 6 months would be questionable from a clinical viewpoint.

## 5. Conclusions

In conclusion, in this histologic monkey study with medium term healing of class III furcation defects no added regenerative effect of PDGF could be demonstrated compared to bone mineral enhanced GTR treatment regardless of the defect configuration. However, postoperative barrier membrane exposure might have influenced the results. These hypotheses should be investigated in further studies.

## Figures and Tables

**Table 1:** Histometric results in small keyhole class III furcation defects.

Histometric Parameters	GTR (*n* = 5)	PDGF (*n* = 5)	Control (*n* = 2)
Defect height (in mm)	2.2 ± 0.5 mm	2.2 ± 0.6 mm	2.3 ± 0.0 mm
New bone height (in % of defect height)	51 ± 17%	42 ± 30%	77 ± 4%
New cementum height (in % of defect height)	44 ± 27%	42 ± 25%	66 ± 6%
New connective tissue height (in % of defect height)	92 ± 11%	77 ± 37%	96 ± 6%
Epithelium height (in % of defect height)	6 ± 11%	20 ± 39%	3 ± 4%
Defect area (in mm^2^)	3.5 ± 1.2 mm^2^	3.1 ± 0.9 mm^2^	3.5 ± 0.0 mm^2^
New bone area (in % of defect area)	27 ± 18%	23 ± 15%	57 ± 13%
New cementum area (in % of defect area)	5 ± 3%	4 ± 3%	7 ± 2%
New connective tissue area (in % of defect area)	63 ± 16%	55 ± 27%	35 ± 9%
Epithelium area (in % of defect area)	4 ± 7%	19 ± 40%	1 ± 2%
Free bone graft area (in % of defect area)	2 ± 2%	1 ± 1%	0 ± 0%

**Table 2:** Histometric results in horizontal class III furcation defects.

Histometric Parameters	GTR (*n* = 5)	PDGF (*n* = 5)	Control (*n* = 2)
Defect height (in mm)	2.2 ± 0.5 mm	2.3 ± 0.5 mm	2.6 ± 0.01 mm
New bone height (in % of defect height)	57 ± 20%	52 ± 23%	80 ± 2%
New cementum height (in % of defect height)	53 ± 15%	40 ± 30%	87 ± 19%
New connective tissue height (in % of defect height)	93 ± 8%	89 ± 7%	97 ± 2%
Epithelium height (in % of defect height)	5 ± 8%	10 ± 9%	0 ± 0%
Defect area (in mm^2^)	3.6 ± 1.3 mm^2^	4.1 ± 1.4 mm^2^	4.3 ± 0.4 mm^2^
New bone area (in % of defect area)	35 ± 15%	27 ± 15%	55 ± 10%
New cementum area (in % of defect area)	5 ± 2%	7 ± 5%	6 ± 1%
New connective tissue area (in % of defect area)	56 ± 19%	60 ± 17%	40 ± 11%
Epithelium area (in % of defect area)	2 ± 4%	6 ± 4%	0 ± 0%
Free bone graft area (in % of defect area)	1 ± 1%	1 ± 1%	0 ± 0%

## Data Availability

The data presented in this study are available on request from the corresponding author D.W.
